# 

**Published:** 1885-01

**Authors:** 


					JANUARY iaae>.
THE
AMERICAN JOURNAL
io?0 F*o*
DENTAL SCIENCE.
EDITED EY
F. J. S. GORGAS, M. D., D. D. S.
UOLu 18.
THIRD SERIES.
no. 9.
BALTIMORE:
GNOWDEN &. COWMAN
LONDON:
TRUBNER 2c CO., 60 PATERNOSTER ROW.
1883.
V?-?Y>
tfPMUBLE IN ADVANCE.*.
:PIim RUFT) MONTHLY
$2.60 PFR RNNIIM.

				

